# Visual Search Strategies of Elite Fencers: An Exploratory Study in Ecological Competitive Situation

**DOI:** 10.3390/jfmk8030106

**Published:** 2023-07-27

**Authors:** Pierre Bagot, Jean F. Fournier, Thibault Kerivel, Cyril Bossard, Gilles Kermarrec, Guillaume Martinent, Marjorie Bernier

**Affiliations:** 1 Centre de Recherche sur l’Éducation, l’Apprentissage et la Didactique, University Brest, F-29200 Brest, France; pierre.bagot@univ-brest.fr (P.B.);; 2 Laboratoire Interdisciplinaire en Neurosciences, Physiologie et Psychologie: Activité Physique, Santé et Apprentissages, University Paris Nanterre, F-92001 Nanterre, France; 3 Laboratoire sur les Vulnérabilités et l’Innovation dans le Sport, University Lyon 1, F-69622 Lyon, France

**Keywords:** fencing, visual search strategies, competitive situation, eye tracking, elite

## Abstract

This study investigates the visual activity of fencers in conditions resembling official competitions. Previous research in experimental conditions has shown that experts focus on specific areas of the torso and the armed arm to control movement initiation. Eight right-handed fencers (epee: two males, one female; foil: one male; sabre: two males, two females) participated in a simulated competition, wearing an eye tracker during one bout. The findings showed that the main fixation in foil and sabre is the upper torso, while in epee, it is the lower torso. In epee and sabre, the upper torso is viewed about 50% of the time, with three other areas also observed, while in foil, the fixation is totally directed to the upper torso. Additionally, two new areas of interest were identified: the score machine and an area involving fixations other than the opponent. The study found no direct link between visual activity and performance. The visual search strategy varies among weapons, with foil using a gaze anchor or foveal spot and epee and sabre utilizing a visual pivot due to the discipline’s inherent rules. The study also emphasizes that competition-like conditions can disrupt visual activity with external stimuli, possibly affecting performance.

## 1. Introduction

Perceptual-cognitive skills play a crucial role in competitive sport [1], particularly in combat sports (e.g., karate, boxing, fencing), where athletes are constantly anticipating the forthcoming attack of their opponent based on prior cues or information available from the opponent’s behavior [2]. Athletes in combat sports fight at close range and require superior perceptual abilities to adapt to opponents’ attacks [3,4]. In order to anticipate opponents’ attacks, react, and respond with quickness and accuracy, fighters must perceive valuable information from a large quantity of dynamically shifting information about the competition. This information may be derived from a variety of sources, such as the opponent’s movements and posture, the distance to the opponent, or the match’s status [2]. Such information could be recognized and processed by athletes through visual activity, allowing them to foresee the behavior of their opponents and make decisions more suitable to winning the game.

In fencing, an average action to score a point lasts 5 s in foil and 15 s in epee [5], so a quick and adequate reaction to an opponent’s actions is one of the main determinants of performance in fencing. Therefore, for fencers, the method of perceiving information from the environment (i.e., kinematic information from the opponent) plays a crucial part in the effectiveness of the technical and tactical actions. In sport domains, visual activity has been investigated widely over the past two decades. Several meta-analyses [6,7] have indicated that the visual activity of experts differs when compared to novices. Particularly, experts have fewer and longer fixations than novices. The presence of systematic differences in gaze behavior between experts and novices is consistent in studies in different sports. Few studies have focused on the visual search strategies (i.e., combination of different variables: area of interest (AOI), fixation duration, number of fixations, saccades duration) of fencers. One of the first studies came from Hagemann et al. [8] who examined the eye movement of 15 expert fencers, 15 advanced fencers, and 32 sports students. In laboratory settings, using the spatial occlusion paradigm, participants were invited to sit and watch fencing attacks on a computer screen (first-person point of view). After this, the occlusion participants had to click on the anticipated target area. Three eye-tracking variables were recorded: viewing time (dwell time %), fixation duration, and number of fixations in each video clip and on each AOI. The results showed that fencers of all performance levels fixated predominantly on the trunk and on the opponent’s weapon, but expert fencers recorded longer fixations than advanced fencers and sport students on the upper trunk region. Also, novice fencers tended to fixate much longer on the upper legs of their opponent compared to advanced and elite fencers. When the trunk of the opponent was occluded from the clips, all participants changed their visual activity from the trunk to adjacent areas. Advanced fencers and sport students recorded a significant decrease in performance (% prediction), while expert fencers’ performance prediction did not change, demonstrating an expertise-level effect. Another study, where an expert vs. novice paradigm was used, indicated that experts use different visual perception strategies than novices [9]. Nineteen participants separated into two groups (experts vs. beginners) took part in this study. Each participant fenced two opponents, one left-handed and one right-handed. The results showed that novices tend to fixate much longer on the opponent’s weapon compared to expert fencers. Moreover, novices spent an equivalent amount of time looking at five different areas (guard, foil, armed hand, lower trunk, and upper trunk), while experts spent significantly more time on the upper trunk and the armed hand.

Witkowski et al. [10,11] conducted a series of studies on the impact of opponent’s handedness on visual search strategies of fencers, with the hypothesis that experts use different visual search strategies depending on the opponent’s handedness. In the first study [10], 12 expert foil fencers were invited to fight two opponents, one with right-handedness and the other with left-handedness, during 20 s duels. The results showed that when facing left-handed opponents, experts tended to fixate more often and much longer on the armed hand of their opponents than the other areas (guard, foil, armed hand, lower trunk, and upper trunk). Additionally, when facing a left-handed opponent, experts spent an equal amount of time staring at the armed hand and the upper torso. When facing a right-handed opponent, experts fixated more often and spent more time on the upper torso than when facing left-handed opponents. In addition, when facing a right-handed opponent, experts fixated more often and spent more time fixating on the upper torso than any other body area. Another study by Witkowski et al. [11], also using the opponent’s handedness paradigm, was conducted to find out if attacking and defensive actions had an impact on visual search behavior. Twelve female foil experts took part in this research. For each participant, the study involved two tasks, attack and defense, and two conditions, a right- and a left-handed opponent. Each participant performed 10 repetitions of each task under each condition, which altogether amounted to 40 actions. The results showed that during offensive actions, foil fencers spent more time looking at the armed hand and generated a higher number of fixations to this armed hand when facing a left-handed opponent (compared to a right-handed opponent). Moreover, in fights versus left-handed opponents, the armed hand attracted the most fixations compared to other areas of interest (AOIs). The same result was found for defensive actions. During bouts with left-handed opponents, foil fencers spent more time observing and made more fixation on the armed hand. Facing a right-handed competitor makes foil fencers change their visual search strategies. The results showed that the upper torso attracts a higher number of fixations in attack and more fixations and longer observation times on defense than when facing left-handed opponents. Those results were explained by the fact that facing left-handed competitors is less frequent and, thus, they are viewed as less predictable in their actions. These results could be explained by an increase in anxiety that may influence the stimulus-driven attentional system (bottom-up) over the goal-oriented attentional system (top-down) (corresponding with the Attentional Control Theory [12]) and, consequently, may boost the level of attention directed toward threat-related stimuli.

Taken together, these studies help to elucidate the visual search strategies used by expert fencers. Two methodologies were used to investigate these phenomena. The first included an experimental methodology [8], where fencers were sitting in front of a screen and using a joystick to respond. The other one, with more ecological conditions, based on research conducted by Witkowski and colleagues [9,10,11], collected fencer’s visual activity directly during a fight. Nevertheless, the ecological nature of this research should be questioned. Two of the three previously cited studies found limitations in the choice of action to be performed [10] or the duration of the bout [11]. In research conducted in 2018 [10], with the fight duration limited to 20 s, it is possible that the fencer’s activity was influenced by being required to perform an action within a predetermined time frame. In addition, there is no indication of how points are calculated. Is was a legitimate duel that ended when the first fencer reached 15 points, or was there no scoring recorded? This issue has a significant impact on performance due to (i) the intensity with which the fencer engages in the combat and (ii) the cumulative effect of stress on performance. Even though a 2020 study [11] was conducted on a piste, the actions requested were forced by the protocol itself (i.e., 10 offensive actions and then 10 defensive actions against a left-handed opponent and then a right-handed opponent, with a balance between the two conditions). This type of protocol, despite being ecological as it collects data directly from participants in action but in a controlled situation, is different from a real competition. Indeed, in a real bout, (i) the duration of a point can range from under a second (an action is performed immediately after the “aller”) to 60 s or more [5], and (ii) attacking, defensive, or counter-attacking actions are not predetermined and are more likely to be produced in the stream of the duel with power relations at play between opponents.

The present study aimed to expand our understanding of the visual activity of fencers by proposing an ecological protocol, quite similar to what expert fencers experienced during competition. To this end, we intended to examine the visual activity of fencers during a simulated competition. To date, no study has investigated the visual activity of fencers during a simulated competition. Therefore, the aim of the present study was to examine the visual activity of fencers in situ. Specifically, with the support of the aforementioned research, we intended to investigate the possibility of various visual search strategies between weapons and between won and lost points.

## 2. Materials and Methods

### 2.1. Participants

A group of 8 right-handed fencers (epee: 2 males, 1 female; foil: 1 male; sabre: 2 males, 2 females) aged from 20 to 31 years (M = 25.88; SD = 3.87), from the French national team, volunteered to take part in this research. According to the classification of McKay et al. [13], 6 participants can be categorized as world-class athletes (Tier 5) with at least one medal at a major global championship in the last Olympic cycle (2020–2024). Additionally, those participants were ranked between the 5th and 172th place [14]. The last two participants can be categorized as elite/international athletes (Tier 4) with at least two participations at a major global championship in the last Olympic cycle (2020–2024). They were ranked between the 130th and 240th place [14]. The study’s research protocol was carried out in accordance with the international ethical guidelines and data protection conditions. The study was approved by the ethics committee of Nantes University with ID number: 08042021 (8 April 2021). All participants were informed about the procedures of the study and signed the informed consent.

### 2.2. Materials and Measures

All measures were performed with a head-mounted Pupil Invisible Eye tracking device (Pupil Labs^®^, Berlin, Germany), with a sampling of 30 Hz and a recording resolution of 1088 × 1080 pixels. Recording was performed with a OnePlus 8 smartphone (OnePlus^®^, Shenzhen, China) worn in a waist bag and connected to the eye tracker. This system allowed for data collection in an ecological setting during a simulated competition. Pupil Invisible eye tracker was chosen because it could be worn under a fencing mask and did not require calibration [15]. Pupil Player app was used to manage and export the data. This software extracts scene video and visual activity recordings and combines them to create a video consisting of scene video and a cursor, indicating foveal vision activity. Frame-by-frame analysis was performed using Adobe Premiere Pro 2023 (Adobe^®^, San José, CA, USA). Each fixation was defined as the condition in which the eye remained stationary for 100 ms or three frames with a variation tolerance of approximately 1.5 degrees [4,6]. The participants’ visual fields were divided into specific AOI, as outlined and analyzed by Witkowski et al. [11]. The first author carried out an analysis on 10% of the dataset before proposing it to two other researchers familiar with this type of analysis. Disagreements about AOI or delimitation of a fixation duration were discussed with regard to the theoretical ground until a consensus was reached between the three researchers. After validation of the encoding, the first author carried out his analysis, independently, on the entire dataset.

The analysis was carried out using three eye-tracking variables:

1. Fixation duration—the average length of fixation on a given area per point;
2. Fixation count—the mean number of individual eye fixations on a given area per point;
3. Dwell time—the time devoted to a given area per point expressed in percentage points.

### 2.3. Procedure

The study was conducted during a simulated competition that replicated an Olympic competition, specifically focusing on the second day of competition (direct elimination table). To recreate the second day of the Olympic competition, each participant engaged in five 15-point matches within a single day, with a 60 min recovery period between matches (equivalent to T64 to semi-final). The opponents were members of the French Team, ensuring that all matches replicated what fencers experience during real international competitions. Similar to actual tournaments, an official referee informed the fencers about the start and end of each point and enforced the corresponding weapon’s rules. One simulated competition was organized for each weapon and gender: female epee, male epee, male foil, female sabre, and male sabre, with the exception of female foil. Due to the organization of the simulated competition, as well as the setup of the eye tracker and the discomfort experienced while wearing it, eye tracker data collection was conducted only during match 1 and match 3. Each participant was briefed on the procedure ahead of the start of the competition. After setting up the equipment, a three-point calibration was performed to ensure that the auto-calibration remained accurate with the mask on, as the eye tracker could have moved. Then, participants engaged in combat with an opponent on a piste in a well-lit fencing hall. The winner of a fencing match is the first fencer to accumulate 15 points. A point begins when the referee says “Allez” (the French word for “Go”) and ends when one of the fencers scores a point that the referee validates. Following each point, both fencers must return to the center of the piste to engage in the next point. In épée and foil, the match is divided into three three-minute periods; if neither fencer reaches 15 points at the end of the three periods, the fencer with the higher score is declared the winner. In sabre, a halftime break is introduced when one fencer reaches 8 points, and there is no time limit; the winner is the first fencer to reach 15 points.

To avoid moisture from the athlete’s sweat infiltrating the eye tracker, to limit the inconvenience of wearing glasses under a mask, and due to the variability in match duration, recording (points and pauses) lasted between 6 min and 14 s and 30 min and 31 s (M = 16.56 min; SD = 08.54 min).

### 2.4. Data Analysis

**Figure 1:**
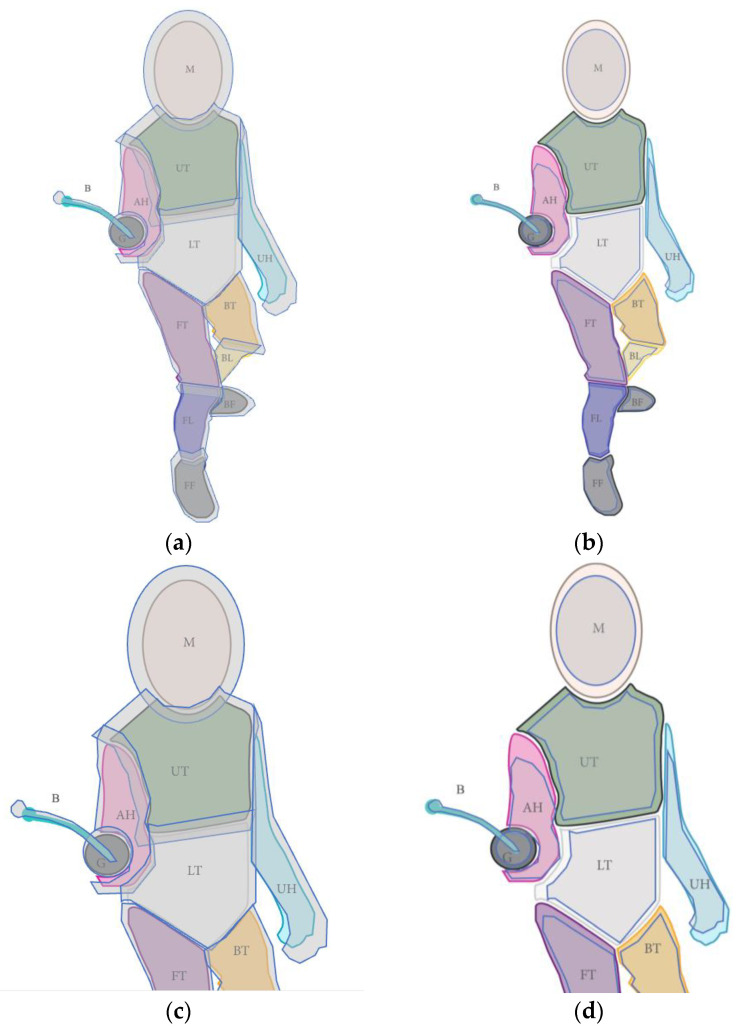
Reference image used during the data analysis. Areas of interest (AOIs) taken from Witkowski et al. [11] and maximum span (represented in red). (**a**) AOI with maximum span for a distance of 300 cm; (**b**) AOI with minimum span for a distance of 300 cm; (**c**) AOI with maximum span for a distance of 100 cm; (**d**) AOI with minimum span for a distance of 100 cm. AH: armed hand; B: blade; BF: back foot; BL: back leg; BT: back tight; FF: front foot; FL: front leg; FT: front tight; G: guard; LT: lower torso; M: mask; UT: upper torso.

The Pupil Invisible (Pupil Labs^®^) Eye tracking device has a constant error margin of 4.5° [15], which represents a deviation of approximately 8 cm when the object fixated is at 100 cm and ~23.6 cm when the object fixated is at 300 cm. Additionally, due to the ecological nature of our research, the distance between fencers constantly varies during the match, so the data must be analyzed with great caution. To do so, the authors developed four figures representing the opponent’s fencer at multiple distances (100 cm and 300 cm) in order to depict the AOIs with the maximum span (see Figure 1). During the analysis, the author compared, when needed, the footage with the appropriate figure to ensure the coding of the right AOI. In addition, gaze motion can help determine where the fencers are fixated. For example, for the blade, only tracking gaze motion can determine whether it is a fixation on the blade or an AOI situated behind the blade, such as the armed hand or torso. Practically, if a fixation was made on the blade and a smooth pursuit followed afterward, we considered it as a fixation on the blade, which extended from the beginning of the cursor stabilization on the blade until the start of the smooth pursuit. Conversely, if the blade moved but the cursor stayed in the same place, fixation was noted to the corresponding AOI. Smooth pursuits were not taken into consideration during the data analysis.

The eye-tracking variables were analyzed separately. Due to the small number of participants to weapons, only descriptive statistics were processed with mean, standard deviation, minimum, and maximum. All the collected data allowed us to extract 267 points, which corresponds to an average of 26 points per participant (min = 15; max = 70). The presented results include fixation duration, number of fixations, and dwell time per point. In addition, to determine if an AOI can be considered as such, a selection criterion of 5 fixations per fencer during the whole experiment was applied.

## 3. Results

The results are presented in four sections: (i) the identification of “Areas Of Interest”, (ii) the AOIs per point (all three weapons combined), (iii) the AOI differences between weapons, and (iv) the comparisons between won and lost point (all three weapons combined). The means and SD displayed below are those for one point. We believe that it is more interesting for professionals, trainers, and athletes to report visual activity on a single point.

### 3.1. Area of Interest

**Figure 2:**
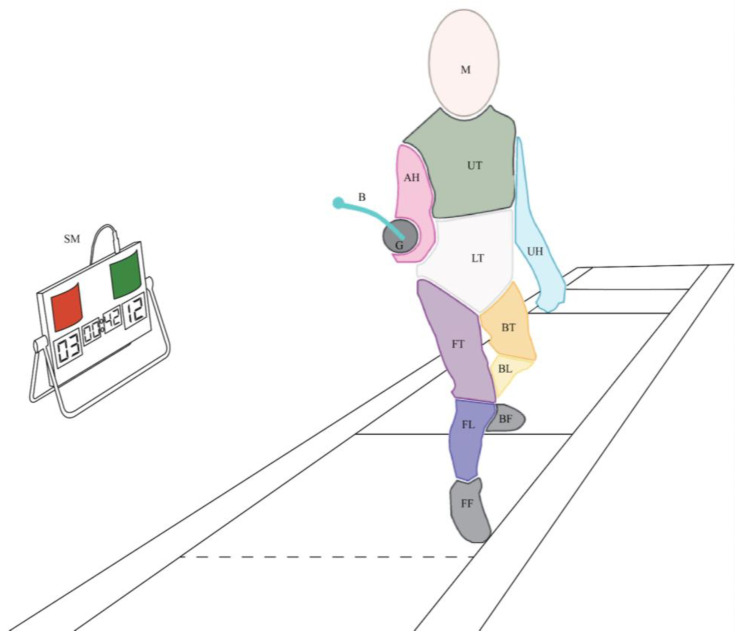
Reference image with areas of interest (AOIs) taken from Witkowski et al. [11] and added AOI: score machine. AH: armed hand; B: blade; BF: back foot; BL: back leg; BT: back tight; FF: front foot; FL: front leg; FT: front tight; G: guard; LT: lower torso; M: mask; SM: score machine; UT: upper torso.

The analysis revealed 9 AOIs already highlighted by Witkowski: armed hand, blade, front foot, front leg, front thigh, guard, lower torso, mask, and upper torso. Two new AOIs were additionally identified: score machine and out of bound; they were not directly related to the opponent (see Figure 2). The first, score machine (SM), was a device that displayed the match score as well as the time remaining in the period. Moreover, a light appears whenever a fencer touches their opponent. The out-of-bound (OB) area refers to different fixations made away from the opponents, in particular a luminous device located at the end of the piste and at a height, which displays a color (green or red) as soon as a fencer touches their opponent.

### 3.2. AOI Per Point

#### 3.2.1. Fixation Duration

For fixation duration (Table 1), we noted that, on average, the armed hand (mAH = 1195 ms; SD = 1166 ms), the lower torso (mLT = 2410 ms; SD = 3466 ms), the mask (mM = 1204 ms; SD = 1099 ms), and the upper torso (mUT = 2013 ms; SD = 1718 ms) were the AOIs where fencers looked for the longest duration during a point. Moreover, when emphasizing on maximum, we can observe that the guard (MaxG = 11,094 ms), the lower torso (MaxLT = 23,451 ms), and the upper torso (MaxUT = 11,759 ms) were the AOIs fixated more than 11 s to 23 s in a point.

#### 3.2.2. Numbers of Fixation

For number of fixations (Table 2), we noted that the armed hand (mAH = 0.75), guard (mG = 1.34), lower torso (mLT = 1.73), and upper torso (mUT = 1.15) were the most frequent AOIs fixated by fencers during a point. All other AOIs, except mask (0.2), were fixated less than 0.1 times, on average, by fencers.

#### 3.2.3. Dwell Time

For dwell time (Table 3), the upper torso (mUT = 53.75%) was the AOI where fencers devoted the most time during a point. In other words, during a point, fencers spent half of the time looking at the upper torso of their opponent. The other half was partially distributed between four AIOs: armed hand (mAH = 9.35%), guard (mG = 7.40%), lower torso (mLT = 18.10%), and mask (mM = 10.25%).

### 3.3. Comparisons between Weapons

#### 3.3.1. Fixation Time

First, we noted that blade, front foot, and front leg were only fixated in epee. In foil, the front tight and score machine were not fixated. Regarding fixation duration (Table 4), in epee, the longest fixation was on the lower torso (mLT = 2542 ms; SD = 2916 ms). In foil, the longest fixated AOI was the upper torso (mUT = 2453 ms; SD = 1373 ms). In sabre, the upper torso (mUT = 2167 ms; SD = 1964 ms) and the lower torso (mLT = 2174 ms; SD = 5023 ms) were the longest AOIs fixated.

#### 3.3.2. Number of Fixations

In epee, as seen in Table 5, the most frequently fixated AOI was the lower torso (mLT = 6.97; SD = 7.10), but the guard (mG = 5.21; SD = 7.2) was also fixated on a substantial number of times. In foil, the most frequently fixated area, during a point, was the upper torso (mUT = 1.43; SD = 0.73). It should be noted that other AOIs in foil were fixated on, on average, less than 0.2 times per point. Finally, in sabre, the AOI that was most frequently fixated on, on average, was the upper torso (mUT = 0.84; SD = 0.72).

#### 3.3.3. Dwell Time (%)

Focusing on dwell time (Table 6), we can observe that in epee, during a point, half of the time spent by the fencer fixating on an area was on the lower torso (mLT = 55.8%; SD = 33.8%), followed by three areas: guard (mG = 23.7%; SD = 29.3%), upper torso (mUT = 10.4%; SD = 20.2%), and armed hand (mAH = 8.4%; SD = 15.2%). In foil, the upper torso was where the fencer spent most of his time during a point (mUT = 96.6%; SD = 8.3%). Lastly, in sabre, fencers spent half of their time observing the upper torso (mUT = 57.4%; SD = 44.6%). During the other half, they fixated on different AOIs: mask (mM = 17.6%; SD = 35.6%), armed hand (mAH = 12.6%; SD = 30.1%), lower torso (mLT = 8.6%; SD = 25%), and guard (mG = 2.8%; SD = 10.5%).

### 3.4. Comparisons between Won and Lost Point

#### 3.4.1. Fixation Time

In regard to fixation time (Table 7), on average, fencers during a won point tended to fixate for a longer period of time on the lower torso (mLT = 2159 ms; SD = 3623 ms) and the upper torso (mUT = 2060 ms; SD = 1862 ms) than any other areas. These results were also found in the lost points.

#### 3.4.2. Number of Fixations

Focusing on the number of fixations, a contingency table (Table 8) shows a similar distribution between won and lost points, except for lower torso and the armed hand with 1.4 more fixations on it in the lost points. When focusing on the average number of fixations per point (Table 9), we noted that the guard (mG = 1.33; SD = 3.89), the lower torso (mLT = 1.95; SD = 4.69), and the upper torso (mUT = 1.09; SD = 1.54) were the only AOIs that had, on average, one fixation per point, regardless of the result of the point. Lastly, we can note that the armed hand (mAH = 0.91; SD: 2.49) tended to be looked at almost one time, on average, per point in the lost point.

#### 3.4.3. Dwell Time (%)

For dwell time (Table 10), whatever the result of the point was, fencers, on average, tended to spend half of the time on the upper torso (mUT = 53.7/53.8; SD = 44.7/45.9). The other half seemed to be devoted to four AOIs: the armed hand (MAH = 11.3/7.4; SD = 25.6/23.2), the guard (mG = 8.5/6.3; SD = 21.1/16), the lower torso (mLT = 14.6/21.6; SD = 30.8/34.2), and the mask (mM = 11.1/9.4; SD = 28.5/28.2).

## 4. Discussion

The aim of this exploratory study was to examine the gaze behavior of top-level fencers in ecological settings. More specifically, we wanted to investigate the possibility of various visual search strategies between weapons and between won and lost points. To do so, we examined the visual activity of fencers during a simulated competition. Compared to previous studies [10,11], our results (Table 4, Table 5 and Table 6) seem to show that the visual activity of foil fencers, in a more ecological situation, meaning without temporal constraints or the requirement to initiate a specific attack or defend against a specific attack, may differ. Also, there may be variations in visual activity among different weapons. Additionally, our study did not identify differences in visual activity between won and lost points in fencing (Table 7, Table 8, Table 9 and Table 10). We acknowledge the challenges in drawing conclusions from an exploratory study and the limitations of the results presented here. Nevertheless, the following discussion aims to provide some explanations for the findings in comparison to the existing literature and suggest avenues for future research.

In foil, Witkowski et al. [9] showed that fencers fixated primarily on two AOIs, upper torso and armed hand, and explained this by the fact that attention is directed toward the onset of movement initiation. In our study, foil fencers seemed to fixate for a much longer time (Table 4) and for a higher number of fixations (Table 5) on the upper torso of the opponent in contrast with the other AOIs. Moreover, this AOI was the first and nearly the only one on which a foil fencer fixated during a point, with an observation time of 96.6% (Table 6). This difference in results may be explained by the opponent’s handedness, as already demonstrated by Witkowski et al. [10], who showed that experts in front of right-handed opponents fixated primarily on the upper torso with glances on proximal AOIs, like armed hand, guard, or mask. In contrast, in front of a left-handed opponent, experts tended to equally fixate on the upper torso and on the armed hand. In our study, fencers also fought a right-handed opponent; we observed some glances to this aforementioned AOI but with a number of fixations below 0.2 (Table 5) and a dwell time ranging between 0.3% and 0.8% (Table 6). In this study, top-level foil fencers, tier 5 according to McKay et al. [13], anchored their gaze centrally on the upper torso of their opponents and used peripheral vision to react to attacks from the armed hand, like Hausegger et al. [16] showed with martial arts experts or Witkowski et al. [9,10,11] in fencing. In addition, this AOI can be considered as the gravity center of the foil scoring area [17]. Therefore, fixation on the upper torso enables fencers to monitor the onset of movement initiation (armed hand) and the entire scoring area of the opponent by using foveal and peripheral vision [18].

Secondly, the present study displays some potential differences in visual activity between weapons. Our results indicated that some AOIs were only fixated on one weapon (Table 4, Table 5 and Table 6), like, for instance, all the AOIs located under the lower torso in epee (i.e., front foot, front leg, front thigh). Furthermore, we noticed that, in contrast with the foil fencer, who mainly fixated on one area, in sabre and epee, an average of three to four areas were fixated per point, with a primary fixation on the upper torso in sabre and then mask, lower torso, and armed hand (Table 4, Table 5 and Table 6). For epee, we noted a main fixation on the lower torso and then a distribution between guard, upper torso, and armed hand. This difference in visual search strategies can be explained by the inherent rules of practice. In epee, points can be given when a “touche” is executed on a part of the entire body, in contrast with foil, where only the torso can be touched (“touché”), or in sabre, where only the upper part of the body can be touched (“touché”) or sliced. Consequently, in addition to monitoring the onset of movement initiation, located on the armed hand, fencers need to track all other areas where they can score. To monitor both of these and reduce the cost associated with saccadic eye movement [19], fencers anchor on a central point (i.e., upper torso in sabre, lower torso in epee) and shift between different cues around this pivot point [17]. This shift between locations can be explained by the importance of those areas for scoring and the need to be processed with the fovea to guarantee the possible movement parameterization and execution of movement to this area (quiet eye; for a review see [20,21]).

Thirdly, the findings appear to indicate that visual activity is not related to a gain or a loss of a point (direct performance indicator) (Table 7, Table 8, Table 9 and Table 10). Two hypotheses can be provided to explain these results. The first is that eye tracking enables the collection of data about the activity of foveal vision. It does not characterize the activity of peripheral vision or can only provide an estimate of the peripheral visual field (40° of the visual angle). In addition, during fixation on AOI, two forms of attention may be present. Either the information is processed by foveal vision, in which case, attention and foveal fixation are combined and referred to as overt attention [22], or peripheral vision processes the information, in which case, foveal vision and attentional focus diverge (covert attention) [18]. It is possible that when a fencer looks at a specific area of interest, such as the upper torso, their covert attention may change into overt attention or vice versa, and this could have an impact on the outcome of the point. However, the distinction between these two categories of attention cannot be made based solely on gaze behavior. The second explanation is that the selected participants may not have allowed for the differentiation of visual activity based on performance to be highlighted. Indeed, it is well-established in the scientific literature that, compared to novices, experts exhibit distinct visual activities that underlie different performances [21]. In karate, experts exhibit shorter response time than novices, with fewer fixations of longer duration and fewer locations, compared to novices [23], or in boxing [3], where experts made fewer fixations than novices with fixations mainly directed to the head, whereas novices fixated mainly and longer on the arms and fists, leading to a significant decrement in decision accuracy. However, in this case, with the selected participants (Tier 5 [13]), it is plausible that the difference between points scored and points lost was related to other factors than simple gaze behavior.

Finally, this study, conducted in a simulated competition environment, placed fencers in conditions that aimed to replicate accurately what they may encounter during competitions. Two new areas were identified via the qualitative analysis (AOI) of the visual activity: the score machine and an area referred to as “out of bounds”. The score machine was located centrally, next to the piste. The score and, more importantly, the remaining time are displayed in a table. The eye-tracking data analysis revealed that this region was only fixated upon near the end of a match or just before a pause, when there was just a few seconds left. Fixations on this area—which are not the opponent—could be signs of effective time management but can also be risky. Fencers preoccupied with this area stop focusing on their opponent, which can be detrimental. The second emerged area combines a set of points fixated on by the fencer during the bout that do not target a specific area of their opponent. Even though these fixated areas do not carry information, it seems important to include them. It does, in fact, symbolize a particular visual activity—that of directing attention away from an opponent. This category includes fixations on a light box behind the opponent that turns on a specific color after a touch is scored. In this instance, the fixation enables one to confirm whether they have touched or been touched by an opponent. Other fixations in this category are on details that are irrelevant to the game or the opposition. Although these two areas are not directed towards the opponent, they still report a visual activity performed during a competition and potentially reflect what happens in a real match. Therefore, in situ research seems to be more relevant to depicting visual activity as it is performed during a real competition, in contrast to research that depicts visual search strategies in fencing but in controlled situations [9,10,11] and, therefore, corresponds to a particular situation. It seems important to deliver this information, as it shows that even with experts, top-level athletes, visual activity and, therefore, attention can be disrupted by external elements. These factors should be considered when preparing for major events.

## 5. Conclusions

Among the limitations of our study that must be taken into account when interpreting these results is the difficulty of generalizing from our extremely small and homogenous sample. We can hypothesize that expanding the sample size for future research would be beneficial. This would allow for a more comprehensive understanding of visual activity in fencing, even revealing weapons-specific differences in visual search strategies. Identifying these differences could lead to the development of distinct training methods and strategies that are tailored to each weapon. Another limitation of this study is related to the use of a remote head-mounted eye tracker device. Although remote eye trackers provide a high degree of ecological validity, they have also been noted to have disadvantages [24,25]. Compared to experimental settings where head-mounted eye trackers and chinrests are used, tracking participants’ gaze behavior in less standardized conditions (e.g., when participants’ heads are not restrained) may result in a decrease in data quality, particularly in terms of the amount of lost data and precision of the recorded gaze position [26,27]. Moreover, frame-by-frame analysis of this type of data is difficult. First, the margin of error in the accuracy (deviation) provided by this type of eye tracker can make data analysis more difficult. In other words, the actual point of gaze may differ from the estimated point of gaze, which may lead to misunderstanding the fixed AOI. In addition, the distance between the fencer and his or her opponent can modify the size of the AOI, making it larger or smaller depending on the distance. This limitation leads to a requirement to interpret the results with more caution.

Our study focused on the visual activity during a point; however, future research could investigate the impact of decisions made prior to a point on a fencer’s visual activity. By examining this relationship, researchers can investigate how a fencer’s prior decisions may influence their attentional focus, visual search patterns, and, ultimately, their performance during a match. Additionally, investigating distractor cues and the function of peripheral vision in the management of covert attention will be extremely beneficial. Distractor cues may have a significant effect on an athlete’s performance, so it is crucial to understand how to minimize their effects. Furthermore, examining the role of peripheral vision for covert attention could shed light on strategies that can improve an athlete’s capacity to process pertinent information while maintaining awareness of their opponents.

These various perspectives could be investigated using mixed-method approaches, such as combining quantitative data obtained from eye-tracking technology with qualitative data gathered through self-confrontation interviews, to investigate these phenomena and attempt to explain the links between attention, decision making, and visual activity [28].

## Figures and Tables

**Table 1:** Mean, standard deviation, minimum, and maximum fixation time to particular areas of interest, in a point, expressed in ms.

AOI	Mean	SD	Min	Max
AH	1195	1166	200	7069
B	443	204	234	985
FF	412	148	217	738
FL	268	17	229	316
FT	366	211	134	1005
G	902	1208	167	11,094
LT	2410	3466	158	23,451
M	1204	1099	211	7649
OB	313	209	134	871
SM	323	212	100	768
UT	2013	1718	141	11,759

Note. AH = armed hand; B = blade; FF = front foot; FL = front leg; FT = front thigh; G = guard; LT = lower torso; M = mask; OB = out of bounds; SM = score machine; UT = upper torso.

**Table 2:** Mean, standard deviation, minimum, and maximum number of fixations to particular areas of interest in a point.

AOI	Mean	SD	Min	Max
AH	0.75	2.26	0	16
B	0.03	0.2	0	2
FF	0.04	0.27	0	2
FL	0.02	0.17	0	2
FT	0.04	0.23	0	2
G	1.34	4.12	0	30
LT	1.73	4.51	0	24
M	0.2	0.5	0	4
OB	0.06	0.29	0	2
SM	0.03	0.22	0	2
UT	1.15	1.57	0	13

Note. AH = armed hand; B = blade; FF = front foot; FL = front leg; FT = front thigh; G = guard; LT = lower torso; M = mask; OB = out of bounds; SM = score machine; UT = upper torso.

**Table 3:** Mean, standard deviation, minimum, and maximum time devoted to particular area of interest, in a point, expressed in %.

AOI	Mean	SD	Min	Max
AH	9.35	24.4	0	100
B	0.05	0.5	0	6.5
FF	0.10	0.5	0	4.2
FL	0.00	0.15	0	2
FT	0.45	3.85	0	65.7
G	7.40	18.55	0	98.9
LT	18.10	32.5	0	100
M	10.25	28.35	0	100
OB	0.10	0.8	0	9.7
SM	0.10	0.65	0	9.9
UT	53.75	45.3	0	100

Note. AH = armed hand; B = blade; FF = front foot; FL = front leg; FT = front thigh; G = guard; LT = lower torso; M = mask; OB = out of bounds; SM = score machine; UT = upper torso.

**Table 4:** Mean, standard deviation, minimum, and maximum fixation time to particular areas of interest by weapons, in a point, expressed in ms.

AOI	Epee				Foil				Sabre			
	Mean	SD	Min	Max	Mean	SD	Min	Max	Mean	SD	Min	Max
AH	817	785	200	3622	302	47	268	335	1584	1362	201	7069
B	493	258	234	985	/	/	/	/	/	/	/	/
FF	425	182	217	738	/	/	/	/	/	/	/	/
FL	261	37	229	316	/	/	/	/	/	/	/	/
FT	267	120	134	457	/	/	/	/	888	166	771	1005
G	1117	1756	167	11,094	390	145	201	536	539	396	168	1742
LT	2542	2916	158	16,132	863	529	503	1642	2174	5023	235	23,451
M	344	230	211	609	1139	95	1072	1206	1281	1255	235	7649
OB	245	61	200	334	687	261	503	871	214	56	134	268
SM	363	239	100	768	/	/	/	/	167	NA	167	167
UT	914	649	141	3340	2455	1373	369	5829	2167	1964	267	11,759

Note. AH = armed hand; B = blade; FF = front foot; FL = front leg; FT = front thigh; G = guard; LT = lower torso; M = mask; OB = out of bounds; SM = score machine; UT = upper torso; NA = Not Applicable.

**Table 5:** Mean, standard deviation, minimum, and maximum number of fixations to particular areas of interest by weapons in a point.

AOI	Epee				Foil				Sabre			
	Mean	SD	Min	Max	Mean	SD	Min	Max	Mean	SD	Min	Max
AH	2.60	4.07	0	16	0.06	0.31	0	2	0.21	0.52	0	3
B	0.14	0.44	0	2	/	/	/	/	/	/	/	/
FF	0.18	0.53	0	2	/	/	/	/	/	/	/	/
FL	0.10	0.35	0	2	/	/	/	/	/	/	/	/
FT	0.14	0.44	0	2	/	/	/	/	0.01	0.11	0	1
G	5.21	7.20	0	30	0.16	0.54	0	3	0.13	0.36	0	2
LT	6.97	7.10	0	24	0.08	0.27	0	1	0.13	0.34	0	1
M	0.08	0.41	0	3	0.04	0.20	0	1	0.30	0.60	0	4
OB	0.08	0.33	0	2	0.06	0.31	0	2	0.05	0.27	0	2
SM	0.13	0.42	0	2	/	/	/	/	0.01	0.08	0	1
UT	1.67	2.86	0	13	1.43	0.73	1	4	0.84	0.72	0	4

Note. AH = armed hand; B = blade; FF = front foot; FL = front leg; FT = front thigh; G = guard; LT = lower torso; M = mask; OB = out of bounds; SM = score machine; UT = upper torso.

**Table 6:** Mean, standard deviation, minimum, and maximum time devoted to particular area of interest, by weapons, in a point, expressed in %.

AOI	Epee				Foil				Sabre			
	Mean	SD	Min	Max	Mean	SD	Min	Max	Mean	SD	Min	Max
AH	8.4	15.2	0	63.8	0.6	2.4	0	16	12.6	30.1	0	100
B	0.3	1.1	0	6.5	/	/	/	/	/	/	/	/
FF	0.3	1	0	4.2	/	/	/	/	/	/	/	/
FL	0.2	0.4	0	2	/	/	/	/	/	/	/	/
FT	0.2	0.4	0	1.5	/	/	/	/	0.8	7.1	0	65.7
G	23.7	29.3	0	98.9	0.8	3.2	0	20.8	2.8	10.5	0	67.6
LT	55.8	33.8	0	100	0.7	2.7	0	15.9	8.6	25	0	100
M	0.2	0.7	0	5.5	0.8	4.9	0	33.7	17.6	35.6	0	100
OB	0.2	0.3	0	1.3	0.4	1.6	0	9.7	0.2	0.5	0	3.3
SM	0.3	1.5	0	9.9	/	/	/	/	0	0.3	0	3.4
UT	10.4	20.2	0	100	96.6	8.3	63.2	100	57.4	44.6	0	100

Note. AH = armed hand; B = blade; FF = front foot; FL = front leg; FT = front thigh; G = guard; LT = lower torso; M = mask; OB = out of bounds; SM = score machine; UT = upper torso.

**Table 7:** Mean, standard deviation, minimum, and maximum average fixation time to particular areas of interest between won and lost point, expressed in ms.

AOI	Point Result							
	Won				Lost			
	Mean	SD	Min	Max	Mean	SD	Min	Max
AH	1347	1570	200	7060	1043	762	268	3622
B	328	133	234	422	559	276	264	985
FF	318	95	217	404	505	201	267	738
FL	236	9	228	246	299	25	281	316
FT	483	344	134	1005	249	77	184	334
G	600	441	167	2145	1204	1975	201	11,094
LT	2159	3623	158	23,451	2661	3309	246	16,132
M	1191	783	568	3953	1218	1416	211	7649
OB	297	111	200	503	328	306	134	871
SM	244	143	100	387	403	280	167	768
UT	2060	1862	267	11,759	1967	1574	141	7538

Note. AH = armed hand; B = blade; FF = front foot; FL = front leg; FT = front thigh; G = guard; LT = lower torso; M = mask; OB = out of bounds; SM = score machine; UT = upper torso.

**Table 8:** Contingency table for number of fixations to particular areas of interest between won and lost points.

AOI	Point Result	
	Won	Loose
AH	83	116
B	2	7
FF	5	6
FL	4	2
FT	6	5
G	183	173
LT	269	194
M	18	34
OB	8	7
SM	4	5
UT	151	154

Note. AH = armed hand; B = blade; FF = front foot; FL = front leg; FT = front thigh; G = guard; LT = lower torso; M = mask; OB = out of bounds; SM = score machine; UT = upper torso.

**Table 9:** Mean, standard deviation, minimum, and maximum number of fixations to particular areas of interest between won and lost points.

AOI	Point Result							
	Won				Lost			
	Mean	SD	Min	Max	Mean	SD	Min	Max
AH	0.60	2.02	0	12	0.91	2.49	0	16
B	0.01	0.12	0	1	0.06	0.29	0	2
FF	0.04	0.25	0	2	0.05	0.28	0	2
FL	0.03	0.21	0	2	0.02	0.13	0	1
FT	0.04	0.21	0	1	0.04	0.26	0	2
G	1.33	3.89	0	21	1.35	4.36	0	30
LT	1.95	4.69	0	24	1.52	4.33	0	24
M	0.13	0.40	0	3	0.27	0.61	0	4
OB	0.06	0.29	0	2	0.06	0.29	0	2
SM	0.03	0.21	0	2	0.04	0.23	0	2
UT	1.09	1.54	0	13	1.20	1.60	0	12

Note. AH = armed hand; B = blade; FF = front foot; FL = front leg; FT = front thigh; G = guard; LT = lower torso; M = mask; OB = out of bounds; SM = score machine; UT = upper torso.

**Table 10:** Mean, standard deviation, minimum, and maximum time devoted to particular area of interest between won and lost points, expressed in %.

AOI	Point Result							
	Won				Lost			
	Mean	SD	Min	Max	Mean	SD	Min	Max
AH	11.3	25.6	0	100	7.4	23.2	0	100
B	0.1	0.8	0	6.5	0	0.2	0	2.6
FF	0.1	0.6	0	4.2	0.1	0.4	0	3.5
FL	0	0.1	0	1	0	0.2	0	2
FT	0	0.2	0	1.5	0.9	7.5	0	65.7
G	8.5	21.1	0	98.9	6.3	16	0	75.3
LT	14.6	30.8	0	100	21.6	34.2	0	100
M	11.1	28.5	0	100	9.4	28.2	0	100
OB	0.1	0.7	0	6.3	0.1	0.9	0	9.7
SM	0.2	1.1	0	9.9	0	0.2	0	2.3
UT	53.7	44.7	0	100	53.8	45.9	0	100

Note. AH = armed hand; B = blade; FF = front foot; FL = front leg; FT = front thigh; G = guard; LT = lower torso; M = mask; OB = out of bounds; SM = score machine; UT = upper torso.

## Data Availability

Data not available.
